# Association of Hypoglycemia With Incident Chronic Kidney Disease in Patients With Type 2 Diabetes: A Nationwide Population-Based Study

**DOI:** 10.1097/MD.0000000000000771

**Published:** 2015-04-24

**Authors:** Chia-Jen Shih, Yueh-Lin Wu, Yuan-Hao Lo, Shu-Chen Kuo, Der-Cherng Tarng, Chih-Ching Lin, Shuo-Ming Ou, Yung-Tai Chen

**Affiliations:** From the School of Medicine (C-JS, Y-LW, S-CK, D-CT, C-CL, S-MO, Y-TC), National Yang-Ming University, Taipei; Department of Medicine (C-JS, Y-HL), Taipei Veterans General Hospital, Yuanshan Branch, Yilan; Division of Nephrology (Y-LW), Department of Medicine, Taipei City Hospital, Zhongxiao Branch, Taipei; National Institute of Infectious Diseases and Vaccinology (S-CK), National Health Research Institutes, Miaoli County; Division of Nephrology (D-CT, C-CL, S-MO), Department of Medicine, Taipei Veterans General Hospital, Taipei; and Division of Nephrology (Y-TC), Department of Medicine, Taipei City Hospital, Heping Fuyou Branch, Taipei, Taiwan.

## Abstract

This article aims to investigate the long-term risk of incident chronic kidney disease (CKD) in type 2 diabetes mellitus (T2DM) patients with hypoglycemia.

This nationwide, population-based, propensity score (PS)-matched cohort study involved 2 cohorts: a hypoglycemic cohort and a matched cohort without hypoglycemia. Data from 1.3 million patients with newly diagnosed T2DM between 2000 and 2010 were extracted from Taiwan's National Health Insurance Research Database. Hypoglycemic events were collected using inpatient, outpatient, and emergency department diagnoses. Patients aged <20 years and those with previous histories of CKD were excluded. The association between hypoglycemia and subsequent CKD risk in patients with T2DM was examined using Cox regression analysis after PS matching.

During the mean follow-up period of 4.2 years, a total of 15,036 (1.7 %) patients experienced at least 1 episode of hypoglycemia and 15,036 matched controls without hypoglycemia were identified among 906,368 eligible patients. The incidence rates of subsequent CKD were 26.1 and 14.8 events per 1000 person-years in the hypoglycemic and matched cohorts, respectively. The hazard ratio (HR) of hypoglycemia for incident CKD was 1.77 (95% confidence interval [CI], 1.63–1.92; *P* < 0.001). Compared with those without hypoglycemia, HRs for 1 to 3 and ≥4 episodes of hypoglycemia for CKD were 1.65 (95% CI, 1.50–1.81) and 1.75 (95% CI, 1.34–2.29), respectively (*P* for trend <0.001).

Our study supports the association of hypoglycemia with CKD development among patients with T2DM, possibly in a dose-dependent relationship.

## Abstract 2

Supplemental Digital Content is available in the text

## INTRODUCTION

Hypoglycemia is a major potential adverse effect of diabetes mellitus (DM) treatment with glucose-lowering drugs, such as insulin or oral medications. The presentation of hypoglycemia can be mild, involving symptoms such as dizziness, diaphoresis, and/or disorientation, or severe, involving symptoms such as neuroglycopenia, which represents a medical emergency and causes permanent brain damage if not detected and treated early. Hypoglycemia can also activate the adrenergic response, resulting in vascular hypoperfusion, cardiac arrhythmia, or even sudden death.^1–3^

A post hoc analysis of data from the Action to Control Cardiovascular Risk in Diabetes (ACCORD) study^4^ found that hypoglycemia was associated with a higher risk of subsequent mortality, which did not differ between intensive and conservative glycemic control groups. Hypoglycemia appeared to be the main barrier to achieving optimal glycemic targets and clinical benefits in patients with type 2 diabetes mellitus (T2DM). Observational studies have also found that hypoglycemic episodes may elevate the risks of adverse cardiovascular events, such as stroke and coronary artery disease.^5–7^ However, the impact of hypoglycemia on the development of diabetic kidney disease has not been thoroughly examined since T2DM diagnosis.

The prevalence of diabetic kidney disease in the United States did not markedly decreased among diabetes population between 1988 and 2008, despite the widespread use of glucose-lowering and renoprotective drugs (eg, renin–angiotensin–aldosterone system blockers).^8^ This phenomenon may be explained by the hypothesis that the harmful effects of hypoglycemia during the treatment of DM may affect kidney function. With respect to hypoglycemia, it is biologically plausible that it may induce kidney damage through its effects on sympathetic overactivity and oxidative stress.^9,10^ Thus, we conducted a propensity score (PS)-matched nationwide study that included the majority of patients with T2DM in Taiwan from 2000 to 2010 to compare the long-term risk of incident chronic kidney disease (CKD) between patients who reported having at least 1 hypoglycemic event and those who reported no clinically evident hypoglycemic episode.

## METHODS

### Data Source

Taiwan's National Health Insurance (NHI) program, launched in 1995, offers comprehensive medical coverage, including coverage of ambulatory and emergent care, hospital admission, dental care, prescription drugs, examinations, laboratory tests, and interventions. The compulsory NHI currently covers 99% of Taiwan's 23 million residents. This study used data from the National Health Insurance Research Database (NHIRD), maintained by the National Health Research Institutes (NHRI). The NHIRD has been described in detail in the previous studies.^11,12^ For the current study, we used the Longitudinal Cohort of Diabetes Patients dataset, which has been validated by the NHRI for research purposes. This database consists of deidentified secondary data from a random sample of 120,000 patients with diagnosis of incident DM per year, which represent the majority (about 70%) of this population in Taiwan, from 2000 to 2010. This sampling number of patients (120,000 patients per year) was based on a regulation that allows <10% NHI enrollees’ medical data extracted for research purposes. Previous studies have validated the accuracy of DM diagnoses in the NHIRD.^13^ Diseases were defined based on the International Classification of Disease, Ninth Revision, Clinical Modification (ICD-9-CM) diagnosis codes. Owing to the deidentified and secondary nature of data, this study was exempted from full review by the Institutional Review Board of Taipei City Hospital, Taipei, Taiwan.

### Study Design

This population-based observational cohort study aimed to assess the association between clinically evident hypoglycemia and subsequent CKD in patients with DM. It included 2 cohorts: a hypoglycemic cohort and a control cohort without hypoglycemia. We identified all patients with diagnosis of incident DM between January 2000 and December 2010. The diagnosis of DM was defined by a primary discharge diagnosis of DM (ICD-9-CM code 250.x), 2 ambulatory visits with a diagnosis of DM (ICD-9-CM code 250.x), or use of any antihyperglycemic drug. The hypoglycemic cohort comprised all patients with DM whose first episode of hypoglycemia (ICD-9-CM codes 251.0x, 251.1x, 251.2x) required medical assistance in an inpatient, outpatient, or emergency department. The index date was defined as 91 days after the occurrence of hypoglycemia to avoid immortal time bias. The matched control cohort comprised the remaining patients with DM in whom hypoglycemia had not occurred during the study period. As these patients never developed hypoglycemia, index dates were randomly assigned according to those of patients in the hypoglycemic cohort. Patients aged <20 years (n = 89,594) and those with previous histories of CKD or end-stage renal disease (ESRD) (n = 9958) were excluded in the hypoglycemic cohort. The same exclusion criterion was applied to the matched cohort.

For patients in both the cohorts, we extracted all data regarding demographic variables, diagnosis and procedure codes, and drug prescriptions for the period extending from January 1995 to December 2011, and ensured that all individuals had available data for at least 5 years before study inclusion. Baseline information was collected from the 5-year period before inclusion. Sociodemographic data included age, sex, monthly income, and urbanization. Charlson Comorbidity Index (CCI) score^14^ adapted diabetes complications severity index (aDCSI) score,^15^ and other comorbidities that are known to be the risk factors for CKD were examined. In addition to antidiabetic medications, other concomitant medications that could be the potential confounding effect between hypoglycemia and CKD were also considered (Table 1 ).

**TABLE 1:**
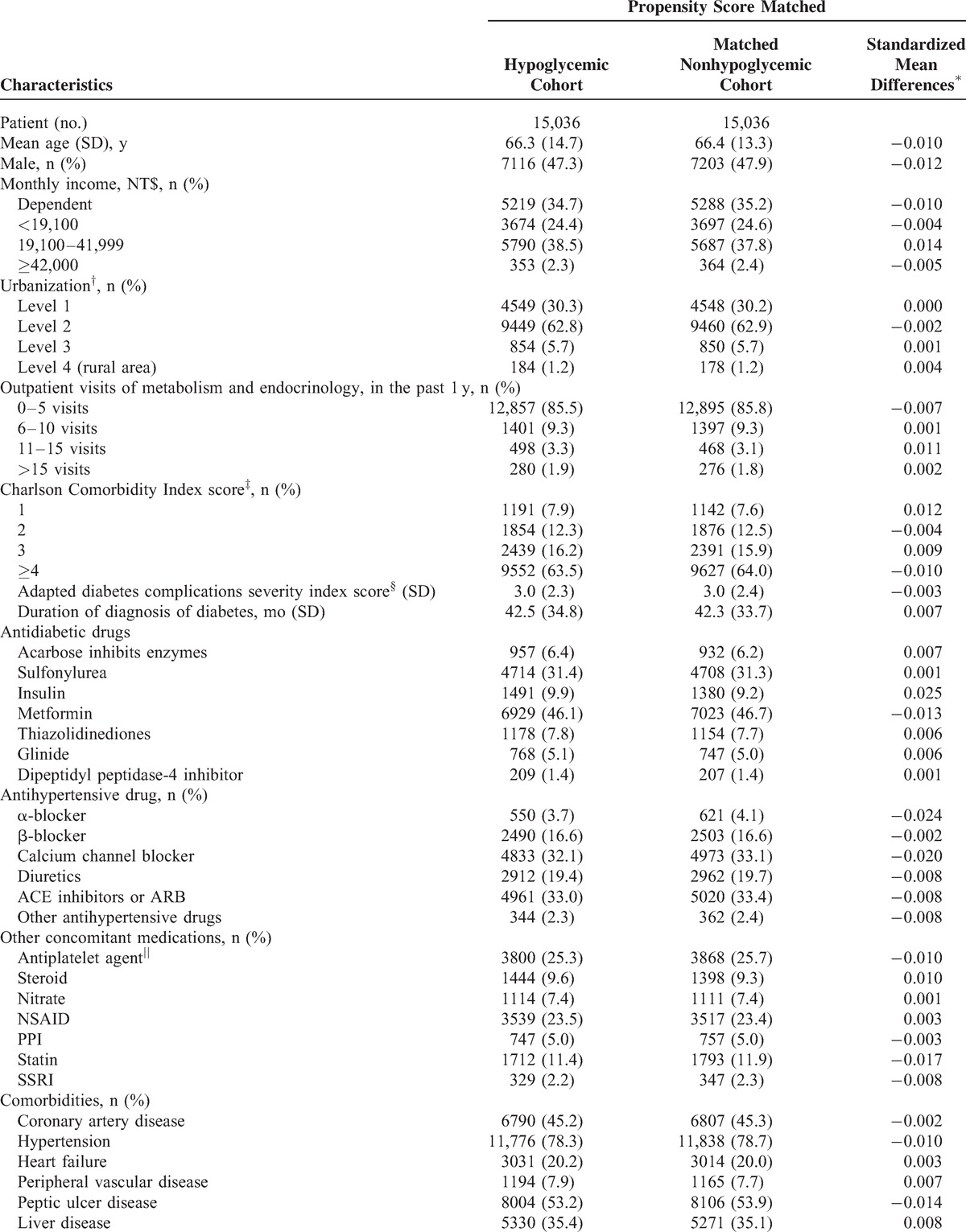
Baseline Characteristics of Diabetes Patients

**TABLE 1 (Continued):**
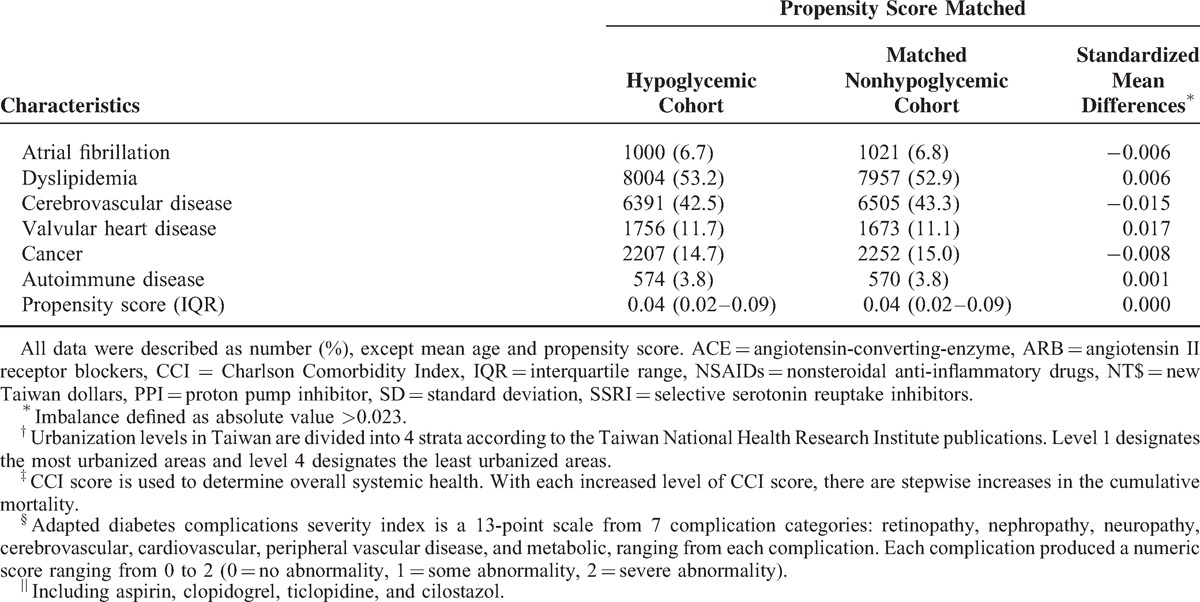
Baseline Characteristics of Diabetes Patients

For each patient, we calculated PSs for the likelihood of hypoglycemia occurrence using baseline covariates and a multivariate logistic regression model. We matched 1 control patient with each patient in the hypoglycemic cohort according to PS (calipers of width equal to 0.1 standard deviation [SD] of the logit of the PS) based on nearest-neighbor matching without replacement.

### Outcomes

The primary study outcome was the occurrence of incident CKD, defined as 2 outpatient visits or 1 hospitalization with ICD-9-CM code 585. This definition was used in a previous study for a similar purpose.^16^ The reliability of ICD-9-CM coding of CKD has also been confirmed.^17–19^ The secondary outcome was all-cause mortality. Both cohorts were followed from the index date until the occurrence of CKD or death, withdrawal from the NHI program, or December 31, 2011.

### Statistical Analysis

Descriptive statistics were used to describe the baseline characteristics of the study cohorts. These characteristics were compared between groups using Pearson χ^2^ tests for categorical variables and independent *t* tests for continuous variables, respectively. Standardized mean difference was used to compare the characteristics between groups after PS matching. PSs for the likelihood of hypoglycemia occurrence were calculated by multivariate logistic regression, conditional on baseline covariates (Supplementary Table 1, http://links.lww.com/MD/A249). The cumulative incidence of CKD was calculated using the Kaplan–Meier method and compared between groups using the log-rank test. Poisson distribution was used to compare the incidence rate of CKD between groups. The relative risk of CKD and all-cause mortality between cohorts was calculated using the hazard ratio (HR) in a Cox regression model. The competing-risks regression was also calculated based on the model by Fine and Gray.^20^ Subgroup Cox regression analyses were performed according to prespecified variables including age, sex, CCI score, hypertension, cerebrovascular disease, myocardial infarction, and insulin use. Interaction tests were performed using the likelihood ratio test.

As at least 3-month observation period was required to diagnose incident CKD, short follow-up period after hypoglycemia occurrence may overestimate the HR for CKD attributed to hypoglycemia. Thus, we performed a sensitivity analysis that excluded patients with follow-up periods <180 and <365 days. We also performed separate analyses dividing the hypoglycemic cohort into 3 subset according to inpatient, outpatient, or emergency department diagnosis of hypoglycemia. Additionally, to test the robustness of our results, we adjusted PS or imbalanced covariates after PS matching. Cox models before PS matching and with the hypoglycemic episode as a time-dependent covariate were also performed. Microsoft SQL Server 2012 (Microsoft Corporation, Redmond, WA) was used for data linkage, processing, and sampling. PSs were calculated using SAS version 9.3 (SAS Institute Inc., Cary, NC). All other statistical analyses were conducted using STATA statistical software (version 13.0; StataCorp., College Station, TX). Statistical significance was defined as a *P* < 0.05.

## RESULTS

### Characteristics of the Study Population

A total of 906,368 patients with diagnosis of incident DM without CKD between January 2000 and December 2010 were enrolled in the study (Supplementary Table 2, http://links.lww.com/MD/A249). Among them, 15,036 hypoglycemic patients and 15,036 matched nonhypoglycemic patients who met the inclusion criteria were identified. The mean age of the study population was 66.6 (SD, 14.3) years. More than half of the patients (52.8%) were female. In the hypoglycemic cohort, the mean interval between hypoglycemia and the first day of DM diagnosis was 42.5 (SD, 34.8) months. The characteristics of the study population are shown in Table 1 .

### Risk of CKD and Mortality

**FIGURE 1:**
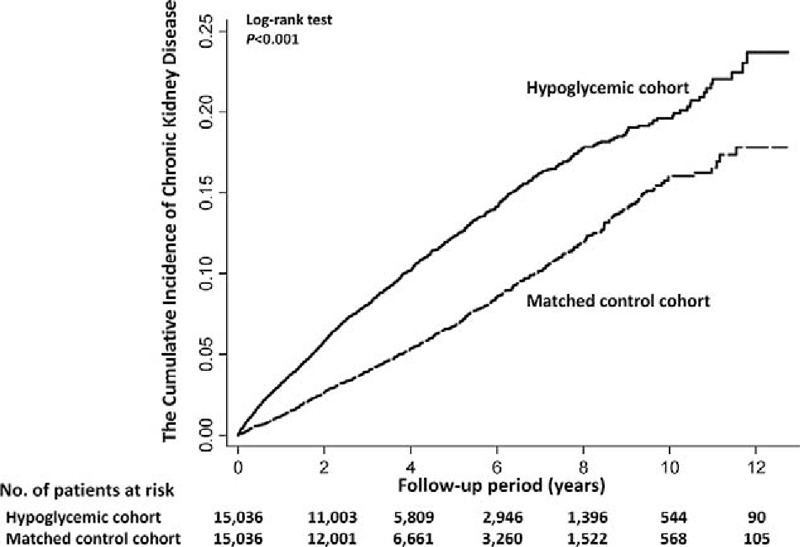
Cumulative incidence of chronic kidney disease compared between diabetes patients with hypoglycemia and individuals without hypoglycemia.

**FIGURE 2:**
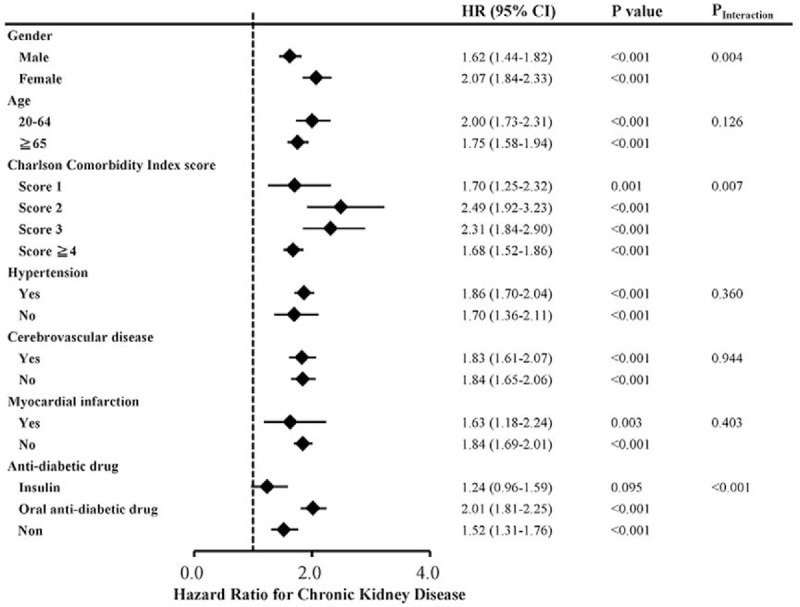
Forest plot of the effect of hypoglycemia on the risk for chronic kidney disease by subgroups.

During the follow-up period, 2419 patients developed CKD and 9776 died before the end of the study. The incidence rates of CKD were 26.14 per 1000 person-years in the hypoglycemic cohort and 14.77 per 1000 person-years in the matched control cohort. The risk of CKD was higher in the hypoglycemic than in the control cohort (HR, 1.77; 95% confidence interval [CI], 1.63–1.92; *P* < 0.001; Table 2). The HR for all-cause mortality among the hypoglycemic cohort was 1.47 (95% CI, 1.42–1.53; *P* < 0.001) compared with the matched control cohort. After considering death as a competing event, the result remained robust (HR, 1.63; 95% CI, 1.50–1.77). The cumulative incidence of CKD in patients with hypoglycemia and the matched cohort was illustrated in Figure 1. Compared with nonhypoglycemic patients, adjusted HRs (aHRs) of CKD for 1 to 3 and >4 episodes of hypoglycemia were 1.65 (95% CI, 1.50–1.81; *P* < 0.001) and 1.75 (95% CI, 1.34–2.29; *P* < 0.001), respectively (*P* for trend <0.001); aHRs of all-cause mortality for 1 to 3 and >4 episodes of hypoglycemia were also statistically significant (*P* for trend <0.001) (Table 3). The association between hypoglycemia and CKD remained consistent in the subgroup analyses. Significant interactions between hypoglycemia and sex (*P*_Interaction_ < 0.001), CCI score (*P*_Interaction_ = 0.043), hypertension (*P*_Interaction_ = 0.015), and using antidiabetic drugs (*P*_Interaction_ = <0.001) were observed (Figure 2). The effect of hypoglycemia on risk of incident CKD was greater in women, patients with low CCI scores, those with hypertension, and those without using insulin (ie, using oral antidiabetic drugs or diet control) compared with those in male gender, high CCI scores, having no hypertension, and using insulin, respectively.

**TABLE 2:**
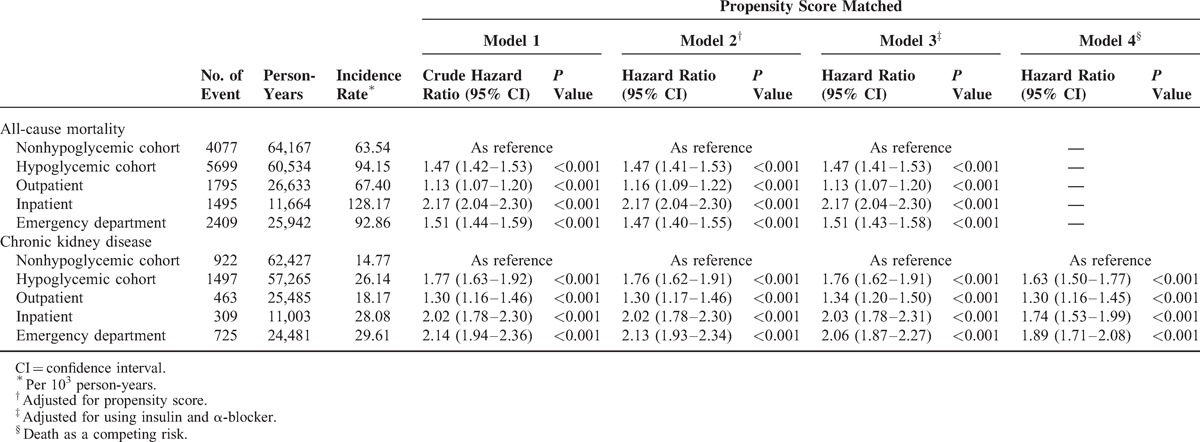
Incidence and Risk of Chronic Kidney Disease and All-Cause Mortality Among Diabetes Patients With and Without Hypoglycemia

**TABLE 3:**
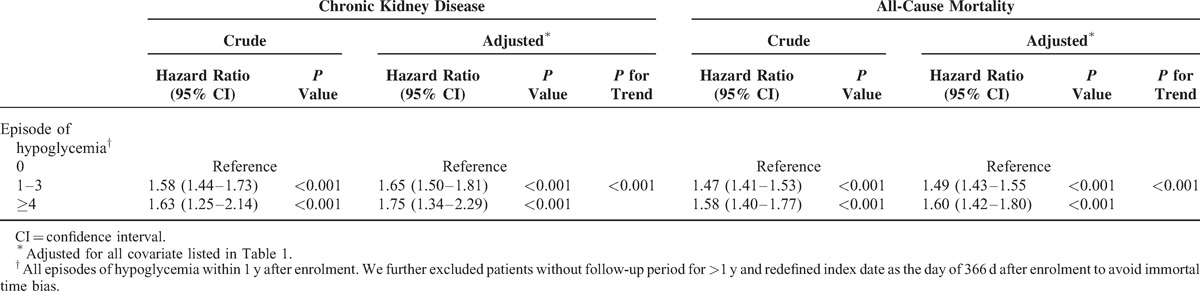
Episodes of Hypoglycemia and Risk of Chronic Kidney Disease and All-Cause Mortality Among Diabetes Patients

Sensitivity analyses that excluded patients with follow-up periods <180 and <365 days confirmed that hypoglycemia increased the risk of CKD in patients with DM (Table 4). The results remained consistent stratified by hypoglycemia diagnosis in inpatient, outpatient, or emergency department (Table 2). Similar results were also obtained in the Cox model with adjusting PS and imbalanced covariates in PS matching (ie, using insulin and α-blockers) (Table 2), before PS matching (Supplementary Table 3, http://links.lww.com/MD/A249), or in the analysis that calculated the hypoglycemia episode as a time-dependent covariate (Supplementary Table 4, http://links.lww.com/MD/A249).

**TABLE 4:**
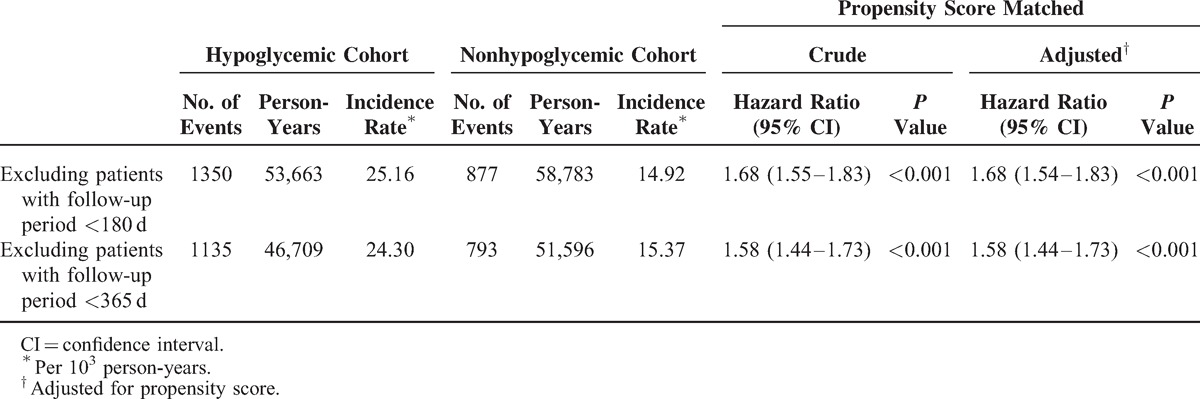
Sensitivity Analysis for the Risk of Chronic Kidney Disease Among Diabetes Patients

## DISCUSSION

In this nationwide cohort of about 1 million patients with T2DM and without CKD at baseline, clinically significant hypoglycemia was associated with a 1.8 and 1.5-fold greater risk of developing incident CKD and all-cause mortality than in matched control subjects without hypoglycemia during the 10-year study period. This association appeared to be dose dependent, as the risk increased with the number of hypoglycemic episodes. The impact of hypoglycemia on incident CKD remained robust after competing risk of death and consistent across subgroups stratified by age, sex, and comorbidities.

Most previous studies^21–23^ have observed increased risks of hypoglycemia and subsequent adverse cardiovascular outcomes in patients with DM and established CKD, possibly due to the prolonged half-life of antidiabetic drugs, which are mainly excreted renally. However, hypoglycemia was also found to be a common complication among patients with DM and preserved kidney function in a cohort of US veterans.^21^ A community-based study found that the risk of severe hypoglycemia was similar across patients with all levels of glycemic control (ie, those with glycosylated hemoglobin [HbA1c] concentrations of <6%, 6% to 6.9%, 7% to 7.9%, 8% to 8.9%, and ≥9%).^24^ The emerging threat of hospital admission for hypoglycemia has also exceeded that for hyperglycemia among older US Medicare beneficiaries susceptible to CKD development or progression.^25^ Intriguingly, small physiological studies have documented acute and transient decline in glomerular filtration rate (GFR) and renal plasma flow during hypoglycemic episodes in healthy humans and patients with T1DM.^26,27^ However, the long-term risk of incident CKD in patients with T2DM who experience hypoglycemia is not completely understood.

A post hoc analysis of Action in Diabetes and Vascular disease: PreterAx and DiamicroN Controlled Evaluation (ADVANCE) study^28^ data showed that severe hypoglycemia was associated with increased risks of microvascular and macrovascular events. However, because of marked attenuation of the association by adjustment for confounding factors and the lack of a dose-dependent relationship in this analysis, the authors suggested that hypoglycemia tended to be a marker of vulnerability to CKD. The influence that patients had untraceable hypoglycemia condition before study entry and the infrequent occurrence of repeated hypoglycemia during the follow-up period may have limited the statistical power to detect the exact effects of hypoglycemia on CKD occurrence in that analysis.^28^ In contrast, the results of our nationwide cohort study suggest that hypoglycemia is a novel risk factor for CKD and a marker presenting with similar severity to that of CKD, as the association between these conditions was dose dependent. This study produced robust and reliable findings because patients received medical attention for hypoglycemia under Taiwan full-coverage NHI program; thus, all significant episodes of hypoglycemia following T2DM diagnosis during the 10-year study period were recorded in the NHIRD. In addition, patients with antecedent hypoglycemia are likely to have attenuated awareness of this condition because of impaired autonomic function.^29^ Thus, the effect of hypoglycemia in a real-world setting may be underestimated, further supporting our findings.

The Kidney Disease: Improving Global Outcomes guidelines encourage the achievement of an optimal glycemic target to prevent or retard the progression of diabetic kidney disease.^30^ However, evidence for the renoprotective effect of glycemic control has been confined almost entirely to the reduction or prevention of albuminuria. Its benefit in terms of clinically meaningful outcomes, such as reduction of GFR decline or ESRD rate, has been examined in only a few studies targeting patients with newly diagnosed DM and no significant cardiovascular risk.^31,32^ Most recent randomized controlled studies, including the ACCORD^4^ study, ADVANCE^33^ study, and Veterans Affairs Diabetes Trial,^34^ have failed to demonstrate remarkably improved outcomes in terms of macrovascular or microvascular complications in patients with T2DM and established cardiovascular risk who received intensive glycemic control. The strategy of intensive control may have exposed patients to increased risks of hypoglycemia and elevated all-cause mortality. Thus, the potential renoprotective benefits of better glycemic control may be attenuated by hypoglycemia during treatment. Although the exact mechanism is not well understood, a preclinical study demonstrated that hypoglycemia can induce nonesterified fatty acid elevation in adipose tissue, which is subsequently associated with kidney damage.^35^ In addition, hypoglycemia could induce sympathetic surges, altering renal hemodynamics^10^; acute fluctuations in blood glucose levels also trigger oxidative stress, which may be responsible for renal function impairment.^9^

Our study has several strengths. First, it examined 1 of the largest cohorts of patients with T2DM worldwide, which consisted of 1.3 million patients representing the majority of T2DM population in Taiwan from 2000 to 2010, and thus minimized referral bias. Second, although the incidence of CKD among patients with T2DM was typically low,^36^ the investigation of a large sample of patients with T2DM and without CKD and comprehensive recording of subsequent hypoglycemic events during an extended follow-up period provided sufficient power to examine the temporal association between hypoglycemia and incident CKD. Additionally, as previous studies have suggested that patients who are prone to hypoglycemia tend to be older and have longer DM durations and more comorbid conditions, such as heart, renal, and/or liver disease,^37,38^ we used PS analysis to reduce confounding effects typical of analyses using observational data.

However, some limitations of this study should be addressed. First, the coded diagnosis of hypoglycemia and CKD may introduce misclassification basis in both the cohorts. Given the sample size is large, we believe that the misclassification bias would be nondifferential and biased the results toward the null hypothesis. Second, the administrative claims data allowed the identification of only clinically significant hypoglycemia; the effect of mild hypoglycemia without medical attention was not analyzed. Third, there were significant interactions between covariates (ie, women, CCI scores, hypertension, and using antidiabetic drugs) and CKD, implying that these covariates may affect the HR. Nevertheless, in separate analyses of the populations with and without these covariates, the effects of hypoglycemia for the HR were in the same direction. Fourth, although the diagnosis of hypoglycemia was based on the clinical assessments of physicians in charge and recorded using the ICD-9-CM code, actual glucose levels during hypoglycemia were not recorded in the NHIRD. Fifth, the database did not contain individual-level information such as body mass index, smoking and alcohol habits, family history of kidney disease, and indicators of glycemic control status, such as HbA1c level. A meta-analysis, however, failed to show the renoprotective effect of lower HbA1c level achieved by intensive glucose control.^39^ In addition, we matched cohorts according to aDCSI scores and the use of glucose-lowering medication, including insulin and oral antidiabetic drugs, which may partly reflect glycemic control and lessen the impact of antidiabetic drugs on risk of CKD.^40,41^ Besides, as other registry-based databases, biochemistry data such as serum creatinine or urinalysis were unavailable in the present database, and therefore different CKD stages were not determined. However, the result of validated diagnosis of coded diagnosis of CKD was similar with that of clinical diagnosis based on estimated GFR.^42^ Finally, detection bias may be introduced in ascertainment of CKD outcomes, and consequently hypoglycemia may be simply a marker for preexisting or kidney disease. However, through implementation of nationwide Diabetes Share Care Network established by the government since 2003, annual serum creatinine measurement was included to early detect renal complications. Therefore, the bias derived from CKD surveillance was less likely to influence our results.

In conclusion, this nationwide cohort study indicated that hypoglycemia is associated with an increased risk of CKD in addition to all-cause mortality, which is strengthened by temporality and a dose-dependent relationship. As patients with T2DM and CKD impose a devastating burden in terms of medical costs and reduced quality and length of life, clinicians must weigh the risks and benefits of T2DM treatment to reduce hypoglycemia for further prevention of kidney disease in patients with preserved kidney function.

## Acknowledgments

*This study was based in part on data from the NHIRD provided by the Bureau of NHI, Department of Health, and managed by the National Health Research Institutes. The interpretations and conclusions contained herein do not represent those of Bureau of NHI, Department of Health, and National Health Research Institutes*.
